# Design circular polarized antenna at ISM band for WBAN using parasitic elements

**DOI:** 10.1016/j.heliyon.2024.e27780

**Published:** 2024-03-15

**Authors:** Muthukumara Rajaguru Kattiakara Muni Samy, Abhishek Gudipalli

**Affiliations:** School of Electrical Engineering, Vellore Institute of Technology, Vellore, India

**Keywords:** Circular polarization, Parasitic elements, Characteristic mode analysis, ISM band, Elliptical patch

## Abstract

This work represents a single layer wide band circular polarized (CP) antenna with broadside high gain. The antenna configuration comprises an elliptical patch serving as the main radiating element, accompanied by eight parasitic components positioned on the same plane as the patch. This setup demonstrates an enhancement in the antenna's bandwidth and gain in the broadside direction compared to conventional antennas. A detailed analysis of the significant modes, using the characteristic mode analysis (CMA) approach, has been employed to optimize the antenna. This optimization has resulted in a notable increase in the 3-dB axial ratio (AR) bandwidth and radiation gain in the broadside direction, attributed to the presence of extra harmonics and the improved aperture efficiency of the parasitic elements. The significant modes are excited via a full-wave electromagnetic (EM) simulation, utilizing a 50 Ω coaxial feed line in the primary antenna. Furthermore, the proposed antenna's functionality is examined through an analysis based on an equivalent circuit model (ECM). To demonstrate the feasibility of the design approach, an antenna prototype is fabricated on a low-cost FR4 material, occupying an overall volume of ${0.58\;\lambda }_{o}\times {0.58\;\lambda }_{o}\times {0.030\;\lambda }_{o}$ (${\;\lambda }_{o}$ is the center operating. frequency). The measured results demonstrate that the suggested antenna operates within a frequency band ranging from 5485 to 6130 MHz for |S11| −10 dB, and the 3-dB axial ratio ranges from 5680 to 5900 MHz. Moreover, the fabricated antenna demonstrates a high gain radiation of 7–7.05 dBi cover the ISM band for biomedical applications.

## 1 Introduction

1Recent developments in wireless technology have spurred the emergence of wireless body area networks (WBAN). These networks have found extensive use in both medical and nonmedical sectors, generating significant interest in antennas as a central component of WBAN sensory systems [[1], [2], [3], [4]]. A wide range of frequency bands have been found to be appropriate for the development of WBAN (Wireless Body Area Network) communication systems through research and commercialization efforts. The Industrial, Scientific, and Medical (ISM) band is covered by frequencies like 402–405 MHz for MICS (Medical Implant Communication Services), 401–406 MHz, 413–419 MHz, 426–432 MHz, 438–444 MHz, and 451–457 MHz for Med Radio; 433.05–434.79 MHz, 902–928 MHz, 2400–2483.5 MHz, and 5725–5850 MHz for the ISM band; and 2360–2400 MHz for Medical Body Area Network (MBAN) [2,4].

The application of WBANs is widespread in a number of common scenarios, such as direct communication between implanted sensory nodes and an off-body transceiver, intra-node communication between a body-attached master node and an off-body transceiver, and inter-node communication among sensory nodes attached to the body's surface [5]. Wireless telemetry, an essential element, encompasses the assessment of signals emanating from the human body and their subsequent transmission via an RF link to a remote base station, like a computer or phone, situated away from the body. This data is then relayed via the internet to the attending physician or relevant individual, enabling accurate diagnosis and appropriate patient guidance [4]. Antenna technology has garnered increased attention in the healthcare sector, particularly within the realm of wireless body area networks (WBANs). As contemporary lifestyles tend to become more fast-paced, individuals are increasingly vulnerable to insidious diseases, often as a result of neglecting regular health check-ups. In this context, WBAN-based proactive health management devices play a pivotal role in enhancing the overall quality of life. Acknowledging the antenna as the fundamental component of any wireless system, its scope has expanded rapidly, not only facilitating data transmission in close proximity to the body but also delving into the domains of medical diagnostics and rehabilitation [6].

The challenges caused by time-varying orientation mismatch resulting from different body postures and motions need to be addressed in order to improve the communication link's dependability. Furthermore, low efficiency, substantial power loss in bodily tissue, and multipath reflections during transmission in interior environments are challenges faced by implanted antennas. For maximum performance in bio-telemetry, high-gain antennas with circular polarization are required to overcome the orientation mismatch between the transmitter and reception antennas, reduce multipath fading, and improve the bit-error rate during data transmission [4,7]. The literature investigates a number of techniques for obtaining orthogonal modes with a 90-degree phase difference, including truncating corners, using numerous slots with metamaterial, including shorting pins, and utilizing a ring-shaped ground with a Z-shaped radiator [7].

In summary, a wide range of operating capabilities is provided by the antennas listed in Refs. [[8], [9], [10], [11], [12], [13], [14], [15], [16], [17], [18], [19], [20]]. However, a major disadvantage of its many layers design is that it leads to complex antenna architecture and higher production costs. Additionally, the existence of an air gap is responsible for their considerable profile and poor mechanical characteristics. The recent advancements in characteristic Mode Analysis, as detailed reported in Refs. [[21], [22], [23]], showcase the generation of circularly polarized characteristics by exciting two orthogonal modes with a 90-degree phase shift between them. This is achieved through a diagonal reshaping of conventional antennas. However, these antennas exhibit limitations in terms of both bandwidth and gain. Another approach reported in Ref. [24] involves the development of wideband circular polarized characteristics using characteristic mode Analysis. Nevertheless, this design also faces constraints due to its limited bandwidth and the incorporation of a multilayer complex structure. Reference [25] presents work on a dual-band circular polarized antenna utilizing characteristic mode analysis. Despite its advantages, this antenna design exhibits drawbacks such as low bandwidth, lower gain, and not radiates in broadside direction due to partial ground and structure. In the pursuit of addressing these challenges, circular polarized antenna designs utilizing characteristic mode analysis have been explored reported in Refs. [26,27]. While these antennas demonstrate desirable gain, they too are subject to limitations in terms of bandwidth.

In this novel work proposed a high-gain, wideband circularly polarized antenna with a straightforward design, achieving an enhanced 3-dB AR bandwidth using Characteristic Mode Analysis (CMA). The antenna is constructed on a single substrate layer and is fed by a 50Ω coaxial cable. It employs an elliptical patch as the main radiating element, complemented by eight parasitic elements strategically arranged around the radiator. This configuration serves the dual purpose of broadening the operating bandwidth and achieving high gain in the broadside direction. The proposed antenna is well-suited for applications in WBAN as a node-centric solution for wireless communication.

The organization of this study is as follows: Section II details the considerations for antenna design, encompassing the examination of the cavity model and CMA. In Section III, a comprehensive full-wave analysis is employed to excite the significant modes. Section IV involves an analysis of the equivalent circuit model, ensuring the validation of the proposed antenna's electromagnetic performance. Section V presents the fabrication process and thoroughly explores the discussion of the measurement results. Finally, Section VI offers the concluding remarks.

## 2 Design consideration of proposed antenna

### 2.1 Cavity model method based modal analysis

**Fig. 1:**
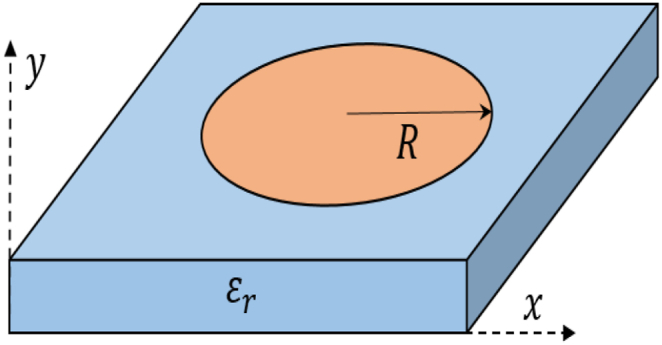
Dielectric-loaded circular patch antenna.

The cavity model method (CMM) serves as an analytical technique used to grasp the fundamental characteristics of conventional microstrip antennas, albeit with crucial assumptions. However, it encounters challenges when applied to irregularly shaped microstrip antennas. The circular cavity model demonstrates several resonance modes. In the case of the $TM_{mno}^{Z}$ mode, the resonance frequency can be expressed as [28]:

(1) $${\left(f_{r}\right)}_{mn0}=\frac{c}{2\pi \sqrt{\varepsilon_{r}}}\left(\frac{\chi_{mn}^{\prime }}{R}\right)$$

here, ‘R’ represents the radius of the circular patch, as depicted in Fig. 1. ‘c’ denotes the speed of light in free space, while ℇ_r_ denotes the relative permittivity of the substrate. Additionally, $\chi_{mn}^{\prime }$ is responsible for determining the order of the resonant frequency. Specifically, at $\chi_{11}^{\prime }=1.8412$ , the first-order mode $\left(TM_{110}^{Z}\right)$ can be determined [29].

Equation (1) can be used to calculate the R parameter of a circular patch antenna. The proposed antenna is designed by *FR-4* substrate with $\varepsilon_{r}=4.3$ and tanδ = 0.025. Its lower frequency for the first-order mode is 5800 MHz ($TM_{110}^{Z}$). The parameter is calculated as given by Equation (2):

(2) $$R=\frac{1.8412*3*10^{8}}{2\pi *5800*10^{6}\sqrt{4.3}}=6.47\;mm$$

### 2.2 Characteristic mode analysis of antenna

The conventional mode analysis (CMA) method finds extensive application in the design of microwave circuits. It offers a systematic, step-by-step design analysis, along with a comprehensive understanding of the physical mode behavior in conducting structures. CMA relies on the Method of Moments (MoM), enabling the numerical calculation of characteristic modes for conducting structures of arbitrary shapes [21,29]. Several factors come into play when conducting a modal analysis of such systems.

1. Eigenvalue $\lambda_{n}$: The eigenvalue corresponding to the nth mode represents the ratio of the reactive stored energy $P_{reac,n}$ near the structure to the radiated energy, $P_{rad,n}$ [29] as given by Equation (3).

(3) $$\lambda_{n}=\frac{<J_{n}^{*}X\left(J_{n}\right)>}{<J_{n}^{\cdot *}R\left(J_{n}\right)>}=\frac{P_{reac,n}}{P_{rad,n}}$$

here, *J*_*n*_ * (where * denotes the conjugate operator) represents the characteristic current, while R and X are the real and imaginary components, respectively, of the impedance matrix corresponding to the conducting body.

The properties of the eigenvalues are as follows [21,[29], [30], [31], [32]]:

(1) $\lambda_{n}>0$, Indicates an inductive mode that stores magnetic energy.
(2) $\lambda_{n}<0$, Indicates a capacitive mode that stores electric energy.
(3) $\lambda_{n}=0$, Indicates the mode is in resonance.
2. Modal Significance (MS_n_): *MS*_*n*_ serves as another critical parameter for representing the radiation characteristics of the mode. It is defined by Equation (4) [30].

(4) $$MS_{n}=\left|\frac{1}{1+j\lambda_{n}}\right|$$

When, $S_{n}>\frac{1}{\sqrt{2}}$, the mode is significant for radiating, and vice versa.

3. The Characteristics Angle (CA), denoted as $\lambda_{n}$ is determined using the following Equation (5):

(5) $$\alpha_{n}=180^{o}-\tan^{-1}\left(\lambda_{n}\right)$$

From a physical standpoint, the Characteristics Angle (CA) signifies the phase disparity between the characteristic currents. A value of $\alpha_{n}=180^{o}$ indicates resonance for the nth mode. In the inductive mode, CA falls within the range of CA is 90° < $\alpha_{n}$ < 180°, signifying the storage of magnetic energy. Conversely, for the capacitive mode, CA ranges from 180° < $\alpha_{n}$ < 270° indicating the storage of electric energy. The determination of the radiating bandwidth involves analyzing the slope at 180° on the characteristic angle curve [[2], [3], [4], [5], [6], [7], [8], [9], [10], [11], [12], [13], [14], [15], [16], [17], [18], [19], [20], [21], [22], [23], [24], [25], [26], [27], [28], [29], [30]].

#### 2.2.1 Mode analysis of Antenna-1

The operational concept of the proposed antenna, which demonstrates enhancements in wide bandwidth and broadside gain in circular polarized antennas, is exemplified through the analysis of modal significance plots and characteristic angle plots utilizing the CMA method. The design and simulation processes were carried out using the CMA Multilayer Solver within the CST Studio 2018 EM tool.

**Fig. 2:**
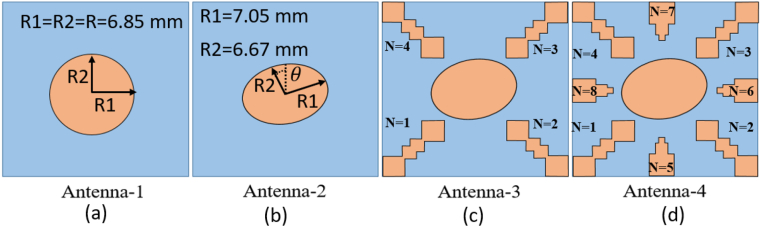
Proposed antenna design steps geometries.

**Fig. 3:**
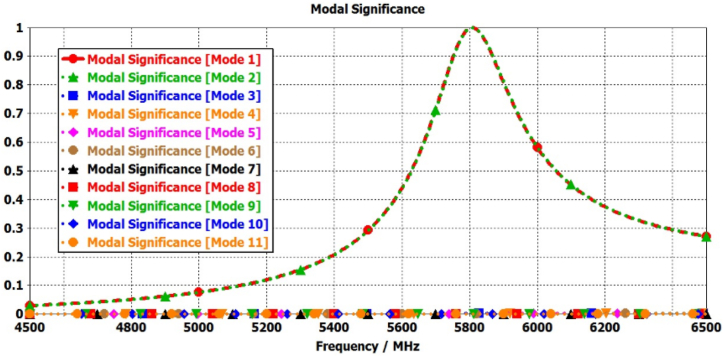
Modal significance plots for Antenna-1.

**Fig. 4:**
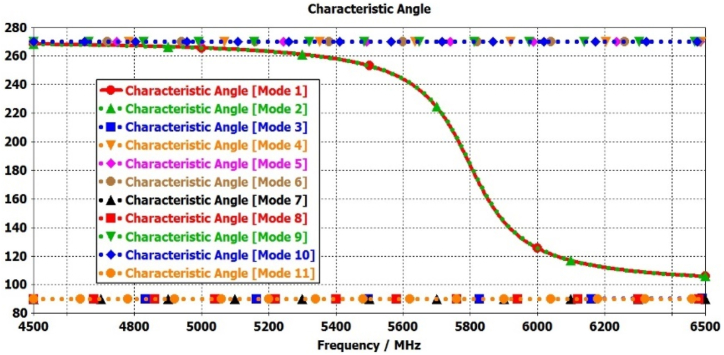
Characteristics angle plots for Antenna-1.

In this step, the initial design of the circular patch antenna is established using the calculated parameter R = 6.85 mm, achieving a resonance frequency of 5800 MHz through the CMA, as depicted in Fig. 2(a). Fig. 3 illustrate the plot of MSn values for eleven modes of antenna-1, where modes 1 and 2 demonstrate resonance due to their MSn values exceeding $MSn>1/\sqrt{2}$ at the 5800 MHz frequency. Conversely, the remaining modes do not resonate due to $MSn<1/\sqrt{2}$. The characteristics of each mode can be analyzed using characteristic angle plots, as demonstrated in Fig. 4. The range of CA for the inductive mode is 90°–180°, indicating that magnetic energy is being stored; for the capacitive mode, the range is 180°–270°, indicating that electric energy is being stored. There is a resonance mode with $\alpha_{n}=180^{o}$ in Modes 1 and 2. Furthermore, Modes 1 and 2 overlap at the same frequency and are orthogonal.

#### 2.2.2 Mode analysis of Antenna-2

To achieve circular polarization in an antenna, two conducting body modes must be present, and these modes should meet specific criteria. The required conditions include [21,[31], [32], [33], [34]].

1. The electric fields of the two modes must be oriented perpendicular to each other on the conducting body. This requirement is verified through the characteristic modes' orthogonality property.
2. The two modes must exhibit identical amplitudes. Consequently, the Coefficient Model Analysis (CMA) technique ensures that the two modes exhibit identical Modal Significance (MSn) values at the specified frequency. This prerequisite is fulfilled through the application of the Model Significance Parameter, denoted as (3).
3. At the designated frequency, the phase difference between the two modes should be precisely 90°. This criterion is verified using characteristic angle parameters, as outlined in (4).

**Fig. 5:**
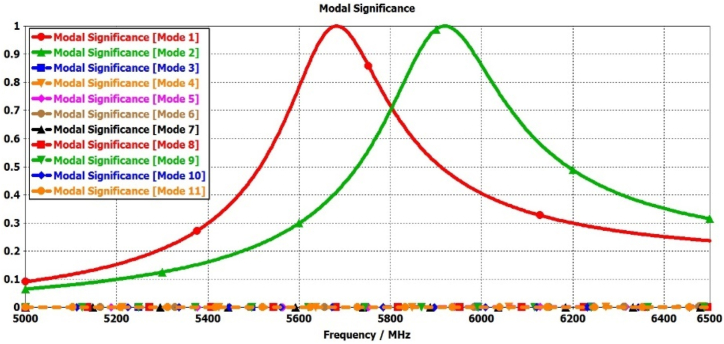
Modal significance plots for Antenna-2.

**Fig. 6:**
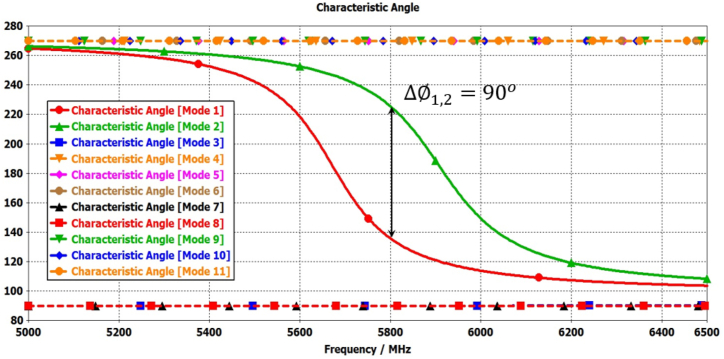
Characteristics angle plots for Antenna-2.

Fig. 2(b), shows an elliptical shape antenna with R1 = 7.05 mm, R2 = 6.67 mm, for 5800 MHz resonance frequency. Fig. 5 illustrates the Modal Significance plot of Antenna-2 at the frequency of 5800 MHz, where both Mode-1 and Mode-2 exhibit an MSn value of 0.7. Moreover, the characteristic angle between the orthogonal modes (Mode-1 and Mode-2) is determined to be 90.45° at the same 5800 MHz frequency as shown in Fig. 6, thus confirming the fulfillment of the circular polarization criteria.

#### 2.2.3 Mode analysis of Antenna-3

**Fig. 7:**
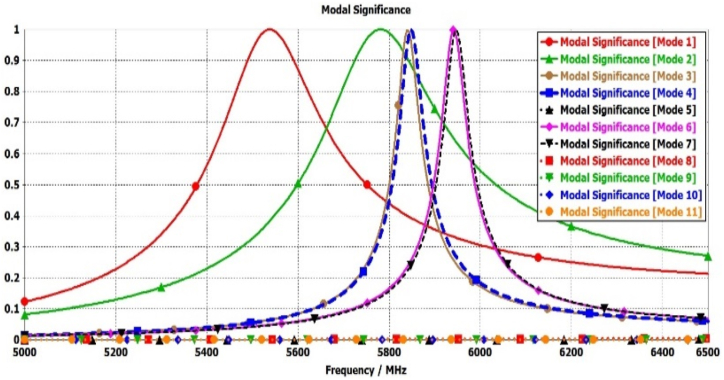
Modal significance plots for Antenna-3.

**Fig. 8:**
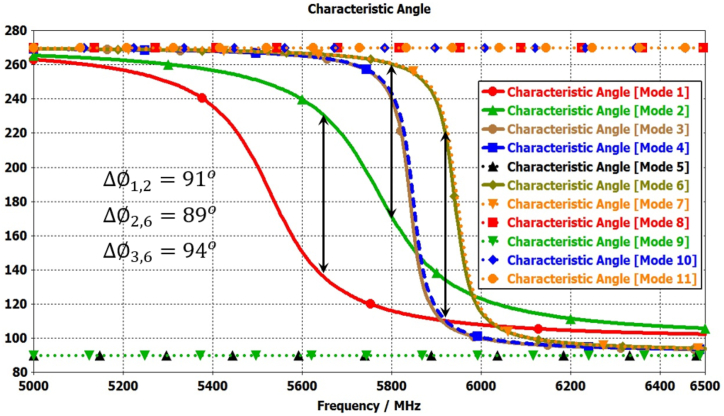
Characteristics angle plots for Antenna-3.

Broadband circular polarization and improved gain can be realized by incorporating parasitic elements surrounding the radiating patch. These parasitic elements are gap-coupled to the radiating antenna, inducing additional modes in conjunction with the patch antenna’ modes [8,22,33,34]. Similarly, broadside gain enhancement is also achievable to amplify the antenna's aperture efficiency [7,35]. Fig. 2(c) represented the elliptical antenna with four similar shape parasitic element (N = 1, 2, 3, and 4) with different gap distance from antenna. The mode-1 and mode-2 are significant for radiation by elliptical antenna and mode-3&4, and mode-6&7 are significant due to parasitic element 2&4 and1&3 respectively as shown in Fig. 7. The characteristic angle differences between mode (1&2), mode (2&3,4), and mode (3,4 & 6, &7) range from 89° to 94°, as illustrated in Fig. 8. These properties are crucial for achieving circular polarization and broadening the bandwidth, owing to the additional modes contributed by the parasitic elements alongside the radiation antenna's modes.

#### 2.2.4 Mode analysis of Antenna-4

**Fig. 9:**
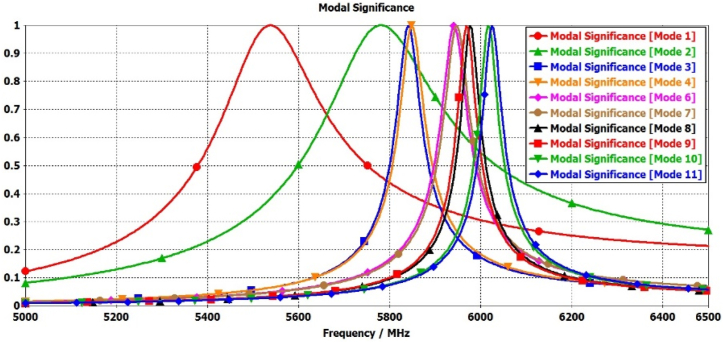
Modal significance plots for Antenna-1.

Furthermore, to enhance broadband circular polarization and broaden the broadside gain, four additional parasitic elements (N = 5, 6, 7, and 8) can be added around the elliptical radiating patch, as depicted in Fig. 2(d). The mode-1 and mode-2 are significant for radiation by elliptical antenna and mode-3&4, and mode-6&7 are significant due to parasitic elements N = 2,4 and N = 1,3 respectively. The mode-8 & 9, and mode-10 & 11 are significant due to parasitic elements N = 6,8 and N = 5,7 respectively as shown in Fig. 9.

**Fig. 10:**
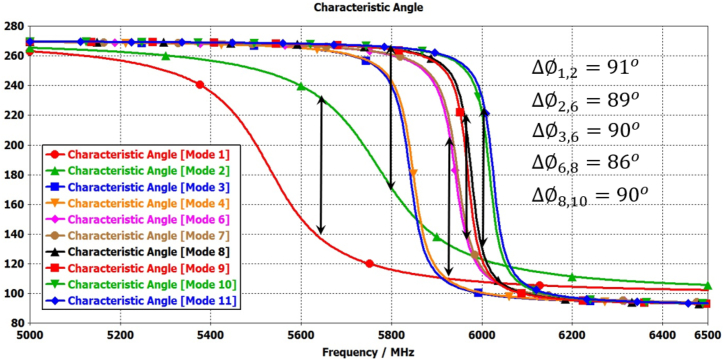
Modal significance plots for Antenna-1.

The characteristic angle differences between mode (1&2), mode (2&3,4), mode (3,4 & 6, &7), mode (6,7 & 8,9), and mode (8,9 & 10,11) range from 89° to 94°, as illustrated in Fig. 10. These properties are crucial for achieving circular polarization and broadening the bandwidth, owing to the additional modes contributed by the parasitic elements alongside the radiation antenna's modes.

**Fig. 11:**
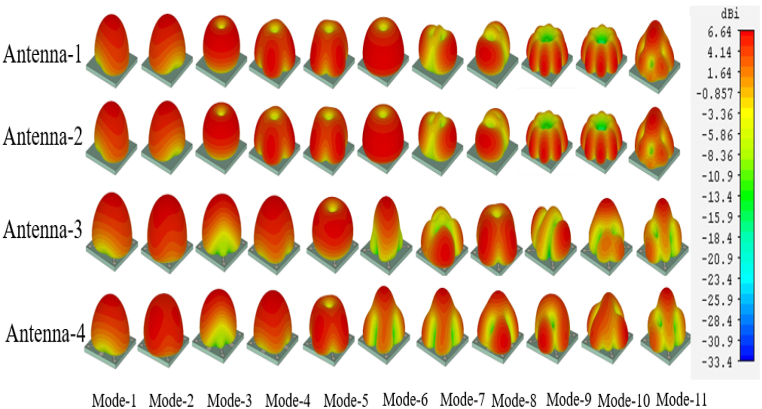
Fairfield radiation patterns for all design steps up to 11 modes.

The enhancement of gain in the broadside direction from antenna-1 to antenna-4 is achieved and the examination of radiation patterns for up to 11 characteristic modes in each step of the antenna design analysis as shown in Fig. 11. Antenna-1 exhibits significant mode-1 and mode-2, both of which display broadside radiation characteristics. The other modes for this antenna demonstrate conical-shaped radiation patterns, which is also the case for antenna-2. Antenna-3 showcases broadside radiation patterns in mode-1 to mode-4 due to the presence of parasitic elements, while mode-5 operates as a magnetic mode. The remaining modes display partially broadside radiation patterns. In the case of Antenna-4, broadside radiation patterns are observed from mode-1 to mode-11, excluding mode-5. The collective impact of all these modes results in the proposed antenna functioning with a broadside radiation pattern and enhanced gain in comparison to conventional antennas. Full Wave Analysis of Antenna with Excitation.

## 3 Full wave analysis of antenna with excitation

**Fig. 12:**
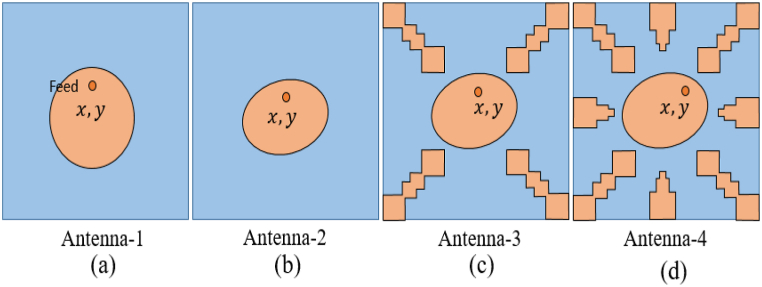
Full wave analysis from Antenna-1 to Antenna-4 using coaxial feed.

**Fig. 13:**
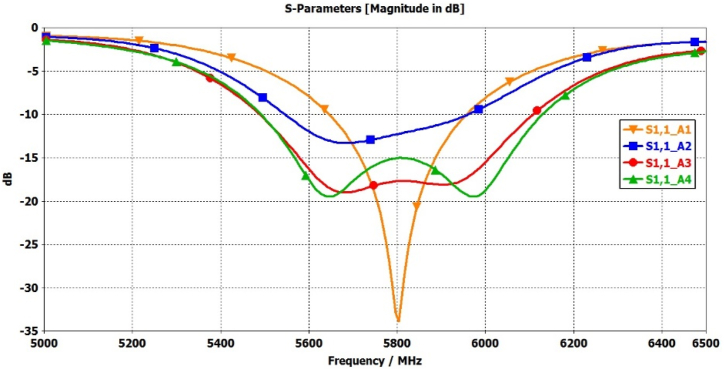
Comparison of S11 parameter from Antenna-1 to Antenna-4.

**Fig. 14:**
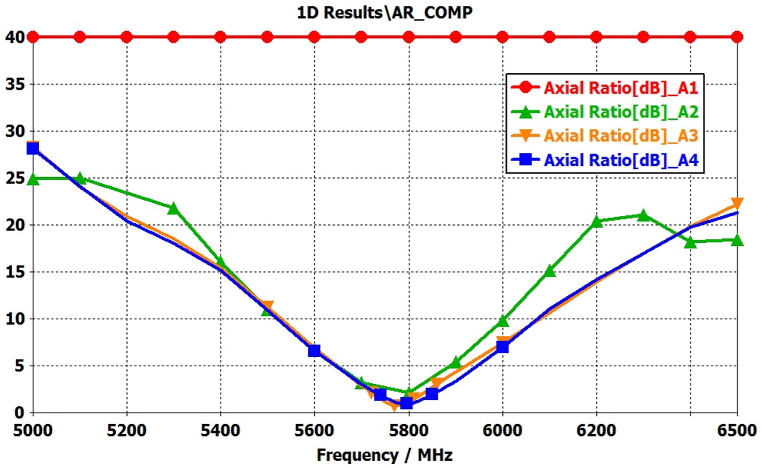
Comparison of axial ratio from Antenna-1 to Antenna-4.

Fig. 12(a–d) illustrates a comprehensive analysis of the significance mode excitation in antenna-1 to antenna-4 using proper feed location. This configuration is designed to achieve a wideband circular polarization, coupled with increased gain in the broadside direction, by appropriately exciting desired modes 1 to 11. In this investigation, a feeding mechanism employs a coaxial probe with an inner-pin diameter of 1.34 mm and an outer-shell diameter of 4.34 mm. The validity of the proposed design is confirmed through full-wave simulations. Fig. 13, Fig. 14 present the simulated results for the reflection coefficient and axial ratio at different stages. Notably, the full-wave simulation results closely align with obtained from the characteristics mode analysis.

**Fig. 15:**
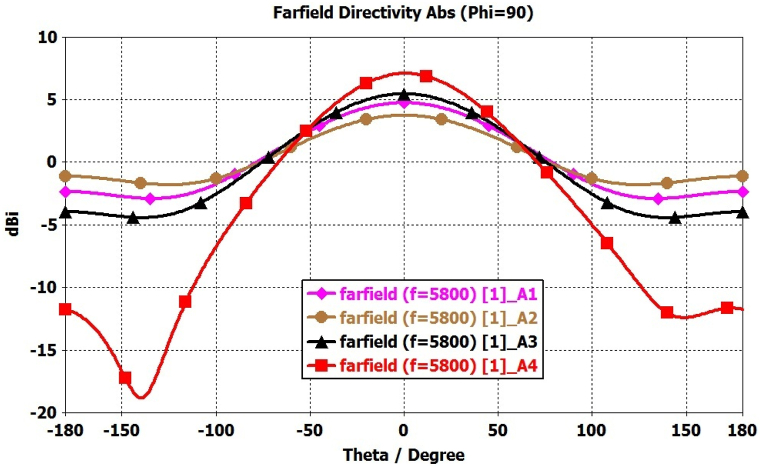
Comparison of Gain(dBi) from Antenna-1 to Antenna-4.

The outcomes demonstrate a significant enhancement in the antenna's performance, particularly in terms of impedance bandwidth (S11 < −10 dB) and circular polarization bandwidth (AR < 3 dB), ranging from 5450 to 6150 MHz and 5625–5950 MHz, respectively. Additionally, the antenna exhibits a noteworthy broadside gain of 7.05 dB at 5800 MHz, as depicted in Fig. 15.

**Fig. 16:**
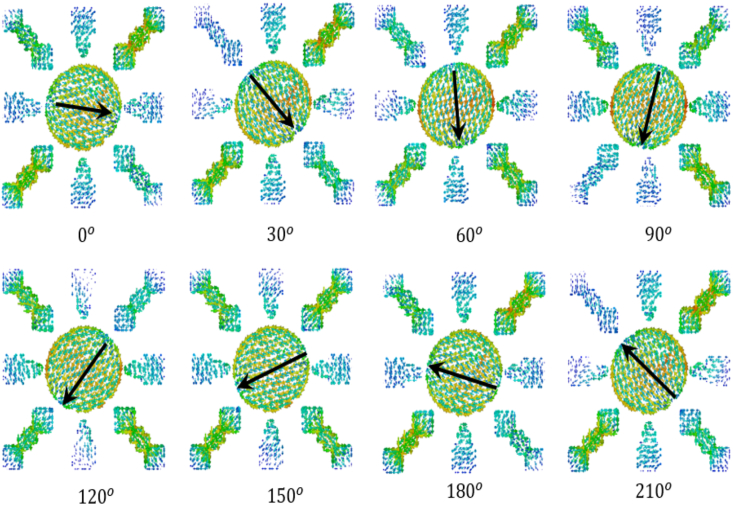
Surface current analysis of proposed antenna with difference phase angle.

**Fig. 17:**
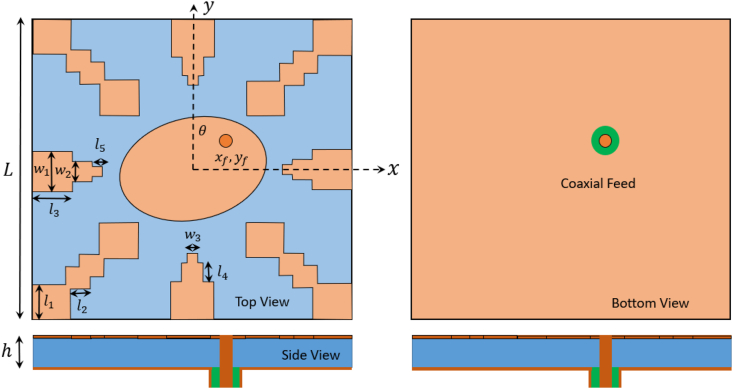
Displays the physically dimensions of the proposed antenna.

Fig. 16 illustrates the surface current distribution of the suggested antenna, depicting varying phase angles. The direction of the surface current is denoted by solid black arrows for each phase angle. Consequently, with different phase angles, we observe the surface rotating in a clockwise direction, indicating its functionality as a left-hand circular polarized (LHCP) antenna. The proposed antenna's dimensions are illustrated in Fig. 17, and the corresponding physical parameters are detailed in Table 1.

**Table 1:** Physical parameters of proposed antenna.

Parameters	Value (mm)	Parameters	Value (mm)
L	30	$l_{1}$	3.2
h	1.6	$l_{2}$	1.66
$w_{1}$	4	$l_{3}$	4
$w_{2}$	2	$l_{4}$	2
$w_{3}$	1	$l_{5}$	1
theta	12 deg.	t	0.035
$R_{1}$	6.9	$R_{2}$	6.20

## 4 Equivalent circuit model analysis of proposed antenna

**Fig. 18:**
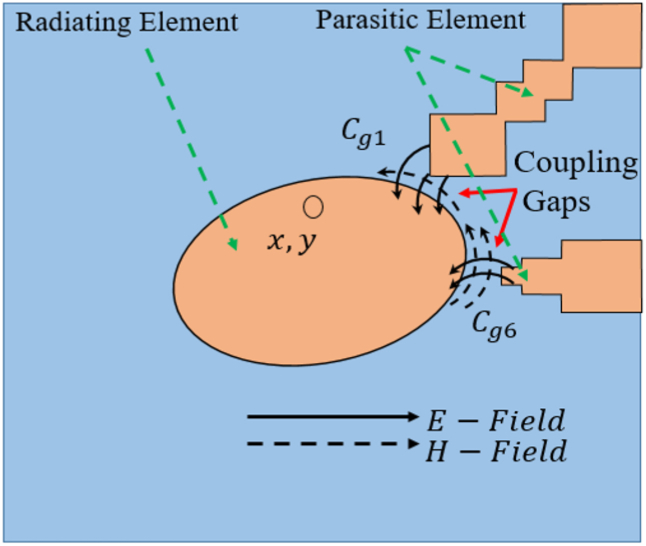
Shows the EM coupling between radiating parasitic element.

**Fig. 19:**
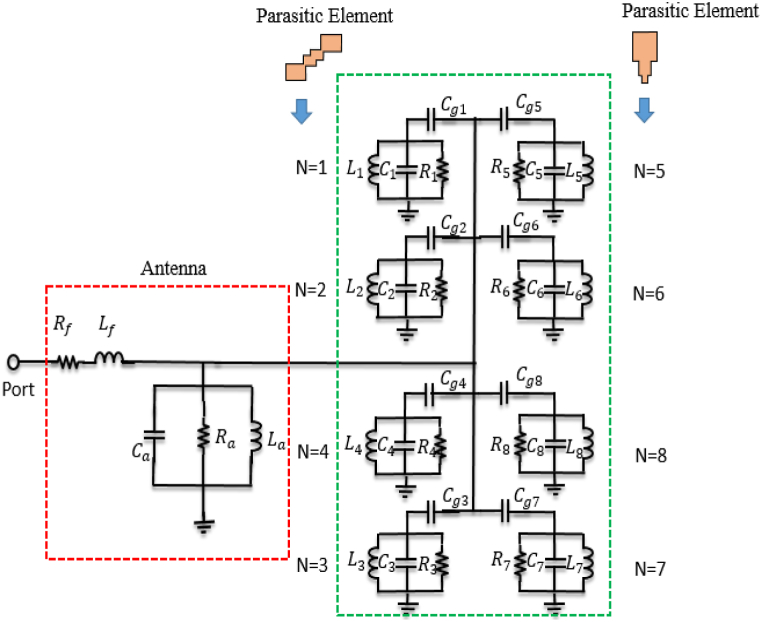
Equivalent circuit modeling of proposed antenna.

**Fig. 20:**
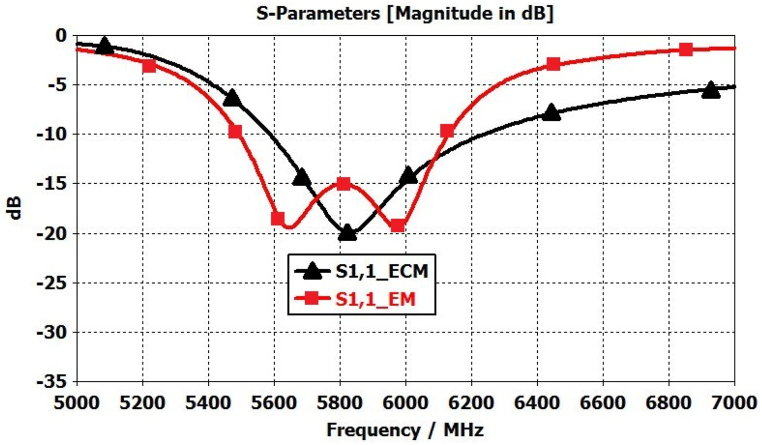
S11 Plots from EM simulated and ECM model simulated.

**Fig. 21:**
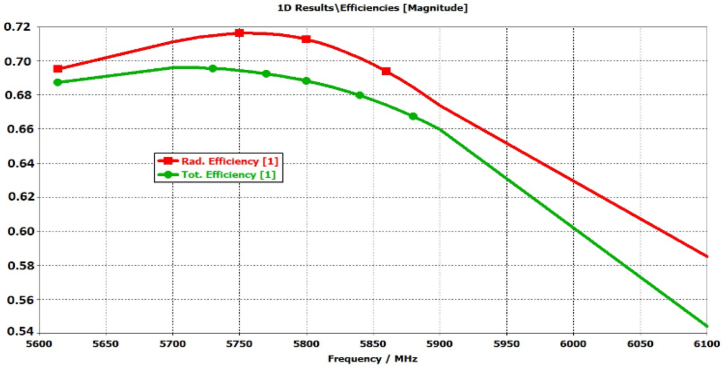
Total and radiation efficiency of proposed antenna.

Fig. 18 illustrates how both electric (E) and magnetic (H) fields can propagate due to the coupling gap between the radiating element and the parasitic elements. This, in turn, leads to the expansion of the antenna's bandwidth and the increase in broadside gain, attributed to the enhanced aperture efficiency of the proposed antenna [23,36]. The electric (E) and magnetic (H) fields guided between the radiating element and the parasitic element can be depicted as running in parallel $C_{g}$ and $L_{g}$ components in an equivalent circuit (with $L_{g}$ disregarded due to its minimal inductive impact) as shown in Fig. 19. Total eight parasitic elements are used in this proposed antenna represented by parallel $R_{N},L_{N},and\;C_{N}$. The radiating elements is represented by of parallel $R_{a},L_{a},and\;C_{a}$. The resonance frequencies of both the radiating element and parasitic elements adhere to the characteristics of a parallel LC circuit. The resonance frequency of the parasitic element pairs (1,3), (2,4), (5,7), and (6,8) is nearly identical due to the uniform gap distance and comparable shapes, leading to the similarity in their electrical parameters. The validation of the equivalent circuit is confirmed by the electromagnetic (EM) simulated S11 results as shown in Fig. 20, and the optimized electrical parameters are detailed in Table 2. The proposed antenna demonstrates radiation and total efficiency exceeding 60% within the operating bandwidth, as illustrated in Fig. 21. The patterns of both curves are approximately identical, with only a few discrepancies. These differences arise from the fact that the equivalent circuit assumes each antenna element operates solely in its dominant mode. However, electromagnetic (EM) analysis reveals that antennas exhibit both dominant and higher-order modes. The higher-order modes give rise to multiple resonance bands when the antenna impedance aligns with the feed line. In this study, the elliptical patch demonstrates two significant orthogonal modes, each featuring two resonance frequencies controlled by adjustments to the radii R1 and R2. When R1 equals R2, both modes coincide, resulting in a single frequency band. The proposed antenna demonstrates radiation and total efficiency exceeding 60% within the operating bandwidth, as illustrated in Fig. 21.

**Table 2:** Electrical parameters of proposed antenna.

Parameter	Value (pF)	Parameter	Value (nH)	Parameter	Value (Ω)
$C_{1}=C_{3}$	1.02	$L_{1}=L_{3}$	1	$R_{1}=R_{3}$	47.5
$C_{2}=C_{4}$	0.95	$L_{2}=L_{4}$	0.92	$R_{2}=R_{4}$	9.15
$C_{5}=C_{7}$	0.94	$L_{5}=L_{7}$	0.19	$R_{5}=R_{7}$	32.5
$C_{6}=C_{8}$	0.11	$L_{6}=L_{8}$	0.94	$R_{6}=R_{8}$	6.72
$C_{g1}=C_{g3}$	1.01	$L_{f}$	0.10	$R_{f}$	49
$C_{g2}=C_{g4}$	1.02				
$C_{g5}=C_{g7}$	0.95				
$C_{g6}=C_{g8}$	0.91				
$C_{a}$	0.01	$L_{a}$	3.95	$R_{a}$	45.45

## 5 Proposed antenna hardware fabrication and measurement

**Fig. 22:**
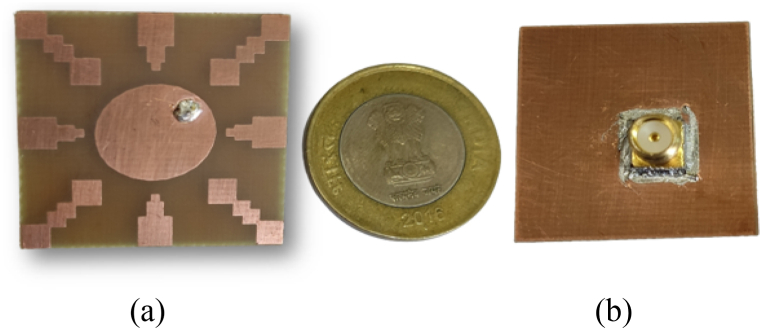
Fabricated hardware prototype. (A) top view; (B) bottom view.

**Fig. 23:**
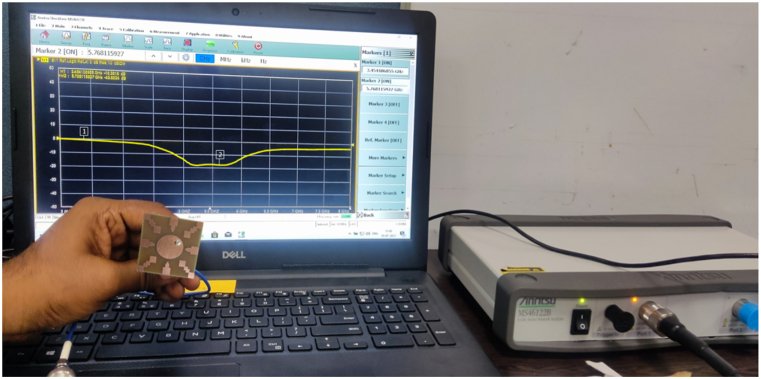
S11 measurement setup using VNA.

**Fig. 24:**
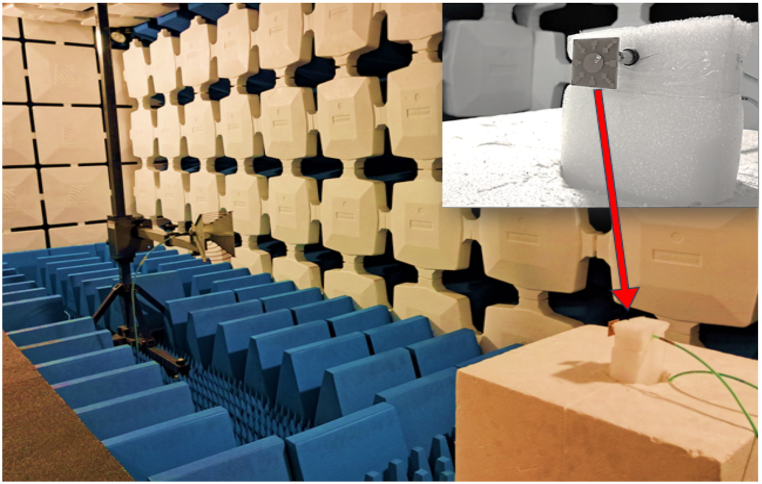
Presents the radiation pattern measurement of the proposed antenna in an anechoic chamber.

The proposed antenna is simulated using the CST Studio suite and was fabricated on a copper-clad FR-4 epoxy substrate (with $\varepsilon_{r}=4.3$ and tanδ = 0.02), illustrated in Fig. 22(a and b), showcasing both the front view and bottom view of the fabricated antenna. To authenticate the design performance, a Vector Network Analyzer (VNA) was utilized for measurement, as depicted in the setup with the proposed antenna in Fig. 23. Additionally, the far-field characteristics of the antenna, such as gain and radiation patterns, were measured in an anechoic environment and are shown in Fig. 24.

**Fig. 25:**
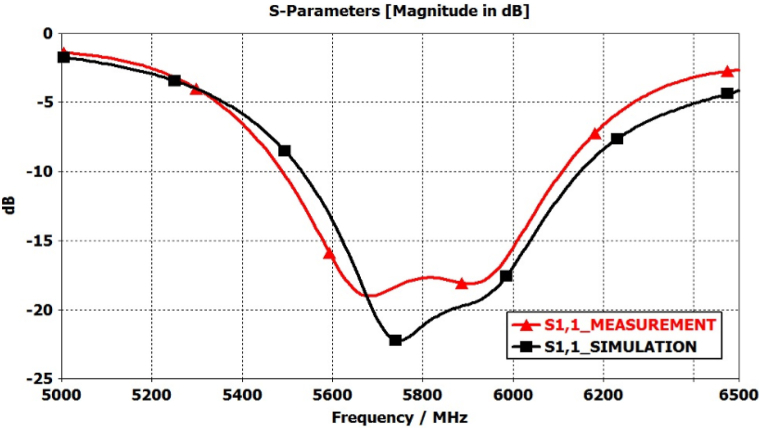
Simulated and measured reflection coefficient of proposed antenna.

**Fig. 26:**
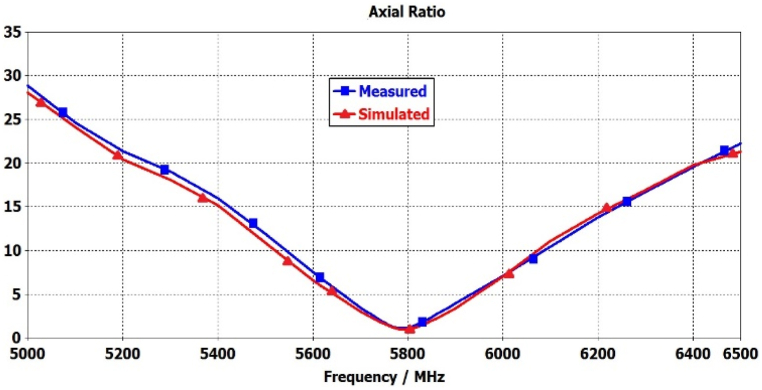
Simulated and measured axial ratio of proposed antenna.

**Fig. 27:**
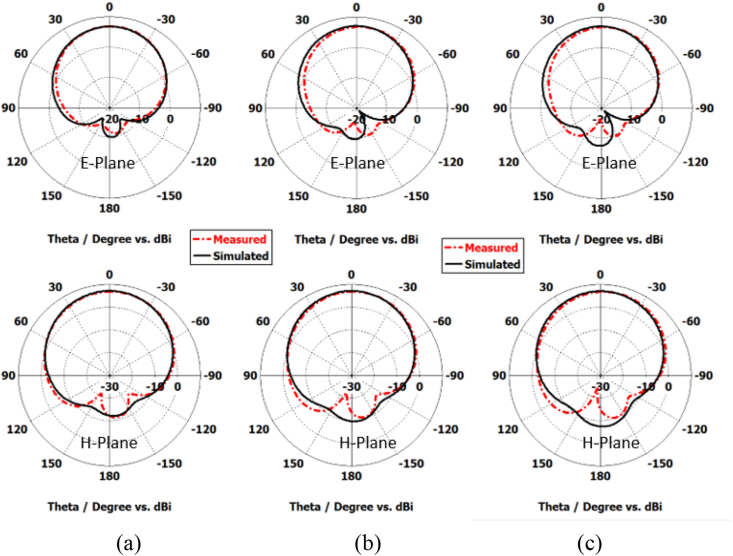
Simulated and measured radiation patterns at (a) 5650 MHz, (b) 5800 MHz, and (c) 5950 MHz.

A comparison between the simulated and measured reflection coefficient (|S11|) for the proposed antenna is shown in Fig. 25. The |S11| measurements facilitate a comprehensive evaluation of the antenna's impedance matching and its effective radiation performance within these parameters. Furthermore, Fig. 26 illustrates the simulated and measured axial ratio of the antenna. The measurement outcomes closely correspond with the simulated results, albeit with slight deviations attributable to fabrication and measurement errors. Fig. 27(a, b, & c) displays radiation patterns at 5650 MHz, 5800 MHz, and 5950 MHz in both the XZ-plane (E-Plane) and YZ-plane (H-Plane), a comparison is presented between simulated and measured results. The figures reveal the antenna's stable and broadside radiation pattern. These patterns offer valuable insights into the distribution of electromagnetic radiation in different planes, delivering essential information regarding the antenna's performance. Table 3 provides a comprehensive performance comparison between our proposed antenna and various circularly polarized antennas reported previously. The antennas reported in Refs. [8,[10], [11], [12], [13], [14],^37^] exhibit larger sizes compared to the proposed antenna. Moreover, the antenna mentioned in Refs. [9,13,14,38] demonstrates lower gain in comparison to our proposed design. In terms of axial ratio bandwidth, the antenna reported in Refs. [9,38] displays a narrower bandwidth, while the antenna discussed in Refs. [8,9,12,38,39] has a reduced impedance bandwidth. The circularly polarized antennas reported in Refs. [[35], [36], [37]] do not have broadside radiation characteristics due to the availability of a defective ground structure (DGS). The proposed antenna is designed without using DGS, resulting in a main broadside radiation pattern with a minimum back lobe. The circularly polarized antennas reported in Refs. [[10], [11], [12], [13], [14]] are complex due to the availability of vias, multi-layer construction, and a large number of slots. These antennas have a high manufacturing cost. In contrast, our proposed antenna is designed using low-cost FR4 material with a single layer and in the same plan. This comparative analysis distinctly highlights the superior attributes of our antenna, showcasing a notably larger axial ratio bandwidth, impressive impedance bandwidth, high broadside gain, and a relatively compact physical size. It is noteworthy that the proposed antenna stands out due to its straightforward design approach, low-profile construction, and exceptionally wide bandwidth.

**Table 3:** Comparison of related work.

Ref.	$f_{o}$, GHz	Printed Layers	Overall Size ($\lambda_{o}$)	Gain (dBi)	Impedance BW (%)	3 dB, AR BW (%)	Complexity	Broadside Radiation Patterns
[8]	5.15	1	1.30*1.08*0.02	12.15	4.84	NA	Simple	yes
[9]	5.8	1	0.27*0.27*0.19	2.1	3.97	2.93	Simple	yes
[10]	5.4	2	0.72*0.72*0.07	7.5	43	34	Complex	yes
[11]	5.4	1	0.98*0.98*0.08	8.8	35	17.5	Complex	yes
[12]	5.8	2	0.68*0.68*0.04	7.2	6.6	3.85	Complex	yes
[13]	6	2	0.78*0.8*0.10	6.5	20.4	20.4	Complex	yes
[14]	5.5	2	1.05*0.97*0.07	6.8	28.9	28.9	Complex	yes
[37]	5.6	1	1.5*1.5*0.14	9.8	85.2	46.34	Simple	yes
[38]	1.57	1	0.17*0.16*1.6	3.17	2.9	3.5	Simple	yes
[39]	5.8	1	0.54*0.46*0.03	–	1.7	3.9	Simple	yes
[40]	3.94	1	0.3*0.3*0.007	–	–	83	Simple	no
[41]	6.4	1	0.4*0.42*1.6	3.8	81.25	30.7	Simple	no
[42]	7.12	1	1.18*1.18*1.6	4.15	–	2.8	Simple	no
This Work	5.8	1	0.58*0.58*.03	7.05	11.10	3.8	Simple	yes

AR = Axial Ratio, BW = Bandwidth.

## 6 Conclusion

A single-layer circularly polarized (CP) antenna with impressive broad functioning and high-gain characteristics has been successfully demonstrated in this work. By employing an innovative configuration, which includes an elliptical patch as the primary radiating component and eight parasitic elements situated on the same plane, the antenna's capacity for enhanced bandwidth and improved gain in the broadside direction has been demonstrated, surpassing that of conventional antennas. A thorough investigation of significant modes through the characteristic mode analysis (CMA) technique has guided the antenna's modification, which provided a noticeable improvement in the broadside direction's radiation gain and 3-dB axial ratio bandwidth. Utilizing the proposed method, the antenna exhibits remarkable advantages compared to conventional antennas, such as broadside radiation patterns with improved gain, wide bandwidth, and circular polarization across a wide range without any defective structure. Additionally, the proposed antenna features a low profile due to its single-layer design, single feed, and use of cost-effective FR4 material, making it easily integrable into hardware. Furthermore, the functionality of the proposed antenna was evaluated using an equivalent circuit model (ECM) analysis. To validate the design approach's feasibility, a prototype antenna was fabricated using a cost-effective FR4 material. The experimental results illustrate that the antenna operates within the frequency range of 5485–6130 MHz for |S11| −10 dB, and the 3-dB axial ratio varies from 5680 to 5900 MHz. Moreover, the fabricated antenna demonstrates a high gain radiation ranging from 7.2 to 7.5 dBi, effectively encompassing the ISM band. This highlights its potential suitability for biomedical applications.

## Data availability statement

The datasets generated and analyzed during the current study are available from the corresponding author on reasonable request.

Funding

Not applicable.

## CRediT authorship contribution statement

**Muthukumara Rajaguru Kattiakara Muni Samy:** Writing – original draft, Validation, Software, Methodology, Investigation, Formal analysis, Data curation. **Abhishek Gudipalli:** Writing – review & editing, Validation, Supervision, Project administration, Data curation, Conceptualization.

## Declaration of competing interest

The authors declare that they have no known competing financial interests or personal relationships that could have appeared to influence the work reported in this paper.
